# Effect of individualized PEEP titration guided by intratidal compliance profile analysis on regional ventilation assessed by electrical impedance tomography – a randomized controlled trial

**DOI:** 10.1186/s12871-020-00960-9

**Published:** 2020-02-20

**Authors:** Jonas Weber, Jan Gutjahr, Johannes Schmidt, Sara Lozano-Zahonero, Silke Borgmann, Stefan Schumann, Steffen Wirth

**Affiliations:** Department of Anesthesiology and Critical Care, Medical Center – University of Freiburg, Faculty of Medicine, University of Freiburg, Hugstetter Str. 55, 79106 Freiburg, Germany

**Keywords:** PEEP titration, Mechanical ventilation, Respiratory system mechanics, Gliding-SLICE, Compliance profile analysis

## Abstract

**Background:**

The application of positive end-expiratory pressure (PEEP) may reduce dynamic strain during mechanical ventilation. Although numerous approaches for PEEP titration have been proposed, there is no accepted strategy for titrating optimal PEEP. By analyzing intratidal compliance profiles, PEEP may be individually titrated for patients.

**Methods:**

After obtaining informed consent, 60 consecutive patients undergoing general anesthesia were randomly allocated to mechanical ventilation with PEEP 5 cmH_2_O (control group) or PEEP individually titrated, guided by an analysis of the intratidal compliance profile (intervention group). The primary endpoint was the frequency of each nonlinear intratidal compliance (C_RS_) profile of the respiratory system (horizontal, increasing, decreasing, and mixed). The secondary endpoints measured were respiratory mechanics, hemodynamic variables, and regional ventilation, which was assessed via electrical impedance tomography.

**Results:**

The frequencies of the C_RS_ profiles were comparable between the groups. Besides PEEP [control: 5.0 (0.0), intervention: 5.8 (1.1) cmH_2_O, *p* < 0.001], the respiratory and hemodynamic variables were comparable between the two groups. The compliance profile analysis showed no significant differences between the two groups. The loss of ventral and dorsal regional ventilation was higher in the control [ventral: 41.0 (16.3)%; dorsal: 25.9 (13.8)%] than in the intervention group [ventral: 29.3 (17.6)%; dorsal: 16.4 (12.7)%; p (ventral) = 0.039, p (dorsal) = 0.028].

**Conclusions:**

Unfavorable compliance profiles indicating tidal derecruitment were found less often than in earlier studies. Individualized PEEP titration resulted in slightly higher PEEP. A slight global increase in aeration associated with this was indicated by regional gain and loss analysis. Differences in dorsal to ventral ventilation distribution were not found.

**Trial registration:**

This clinical trial was registered at the German Register for Clinical Trials (DRKS00008924) on August 10, 2015.

## Background

During mechanical ventilation, it is widely accepted that the application of low tidal volume and low driving pressure, i.e., the difference between plateau pressure (P_Plat_) and (positive) end-expiratory pressure (PEEP), protects the lung from the destructive effects of alveolar overdistension [1–4].

**Fig. 1:**
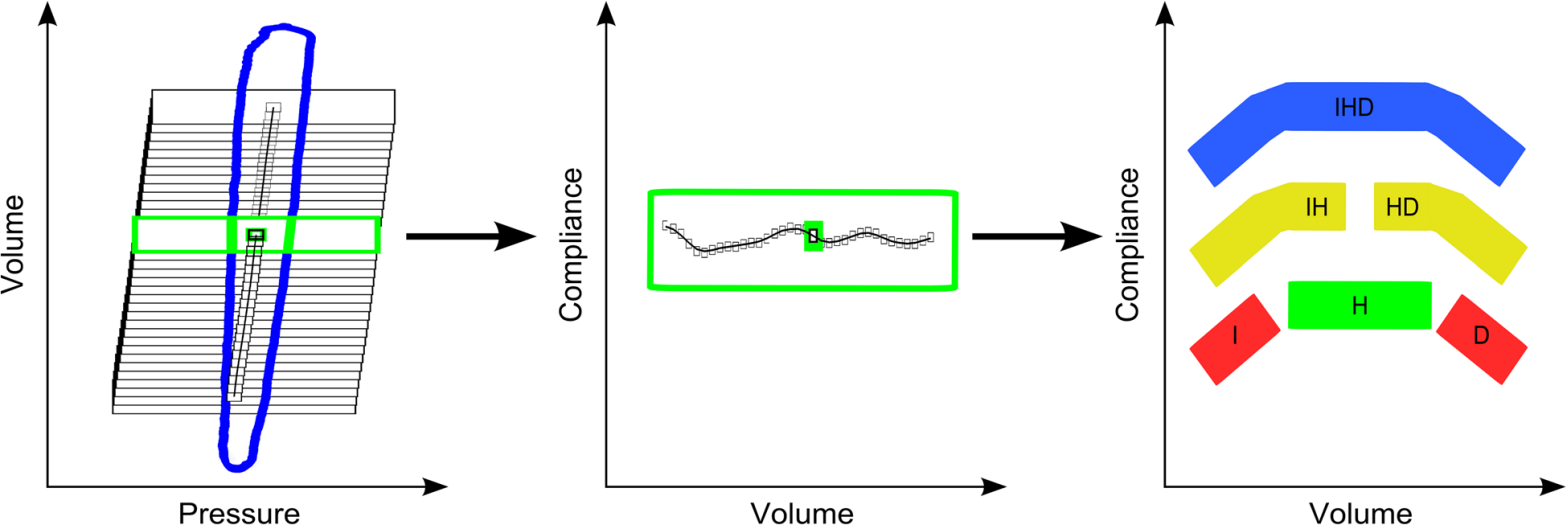
Intratidal compliance profile analysis during a single breathing cycle according to the gliding-SLICE method [20]. The tidal pressure-volume curve is divided into 21 equidistant slices. For each slice, the compliance profile is determined based on multiple linear regression analysis and matched to the respective tidal volume. The resulting intratidal compliance curves were classified into six different compliance profiles (H = horizontal compliance profile, I/IH = increasing compliance profile, D/HD = decreasing compliance profile, IHD = mixed compliance profiles)

With regard to the conflicting clinical data regarding the setting of adequate PEEP during general anesthesia, many techniques have been developed to determine adequate PEEP [5–8]. One technique, first described in 1979 for patients with severe lung injury [9], is based on setting the PEEP slightly above the lower inflection point of the inspiratory limb of the static pressure-volume curve [5, 10, 11]. Other techniques focus on the respiratory system compliance (C_RS_). For example, PEEP can be titrated to reach the maximum quasi-static compliance, calculated by dividing tidal volume (V_T_) by the driving pressure [8, 12, 13]. However, a single compliance value cannot reflect the non-linearity of intratidal respiratory system mechanics during the breathing cycle [14, 15]. To cope with the non-linearity of the intratidal C_RS_ under the dynamic conditions of mechanical ventilation, the gliding-SLICE method [16, 17] was introduced, enhancing the classical SLICE method [18, 19]. To evaluate the intratidal C_RS_ with the enhanced gliding-SLICE method, the pressure-volume curve is subdivided into several volume steps, and the volume-dependent compliance is calculated on the base of data points within a certain volume range (‘slice’) around the current step via multiple linear regression analysis (Fig. 1). The resulting compliance-volume curve can then be classified as follows: an increasing compliance profile is interpreted to indicate intratidal recruitment, suggesting a PEEP increase. A decreasing compliance profile indicates overdistension, suggesting a PEEP decrease. A horizontal compliance profile is assumed to be preferable as, it does not indicate either unwanted condition. Combinations of these three basic compliance profiles may be observed [17] (Fig. 1). A previously described decision support system with a graphical user interface implemented the gliding-SLICE method in a user-friendly tool to recommend at the bedside individualized PEEP titration during fully controlled ventilation [21].

The primary hypothesis of this randomized controlled clinical trial was that individualized PEEP titration, based on analysis of the intratidal compliance profile, would improve the frequency of preferable compliance profiles and ameliorate respiratory system mechanics and regional ventilation during perioperative fully controlled ventilation, compared to a non-personalized PEEP ventilation technique. We determined the frequencies of nonlinear intratidal compliance (C_RS_) profiles and measured regional ventilation, respiratory mechanics and hemodynamic variables in 60 consecutive fully controlled ventilated patients undergoing otorhinolaryngeal surgery.

## Methods

### Ethics, consent and permission

The study was approved by the Ethics Committee of the University Medical Center of Freiburg (vote # 268/15) on June 29, 2015 and registered at the German Register for Clinical Trials (DRKS00008924). This study adhered to the CONSORT guidelines.

### Study design and patient population

After obtaining written informed consent from the participants, we studied respiratory mechanics, hemodynamic variables, and regional ventilation in 60 consecutive patients with American Society of Anesthesiologists (ASA) physical status I-III, who underwent otorhinolaryngeal surgery at the Medical Center of the University of Freiburg, Germany. The study was performed as a prospective parallel-arm, randomized controlled trial with an allocation ratio of 1:1. Randomization was carried out in blocks of 30 by a computer-generated allocation sequence. Participants were enrolled and assigned to the interventions by a study-related anesthetist. The exclusion criteria were ASA physical status > III, age < 18 years, pregnancy, emergency procedure, cardiac pacemaker and other active implants, obesity (body mass index ≥30 kg·m^− 2^), a history of pulmonary disease, laparoscopic surgery, or refusal to participate.

### Procedure

After primary recruitment and preoperative evaluation, the patients received routine monitoring (electrocardiography, SpO_2_ and noninvasive blood pressure measurement; Infinity Delta XL, Dräger Medical, Lübeck, Germany). After preoxygenation to an expiratory fraction of oxygen of 0.8, anesthesia was induced and maintained as total intravenous anesthesia with a continuous infusion of propofol (Propofol 1%; Fresenius Kabi, Bad Homburg, Germany; target-controlled infusion, effect site target concentration for induction: 6–8 μg·mL^− 1^; effect site target concentration for maintenance: 3–5 μg·mL^− 1^, Agilia, Schnider Model; Fresenius Kabi) and remifentanil (TEVA GmbH, Ulm, Germany; induction: 1–2 μg·kg^− 1^, maintenance: 0.15–0.3 μg·kg^− 1^·min^− 1^). During the study protocol, a Bispectral Index™ (BIS™) monitoring (Medtronic, Minneapolis, USA) was used as an additional monitor of anesthesia depth (BIS value target 40–60). Tracheal intubation was facilitated with 0.15 mg·kg^− 1^ predicted body weight [22] iv cisatracurium (Fresenius Kabi). Potential hypotension, defined as mean arterial pressure < 65 mmHg, was treated with a continuous norepinephrine infusion (0.03–0.2 μg·kg^− 1^·min^− 1^). Volume requirements were addressed individually, according to clinical judgement, with a crystalloid solution (Jonosteril; Fresenius Kabi). For tracheal intubation, we used tracheal tubes with low pressure cuffs, with an internal diameter of 7.0–7.5 mm for women and 8.0 mm for men (Mallinckrodt Hallo-Contour; Covidien, Neustadt an der Donau, Germany). All patients were ventilated in the volume-controlled mode with a tidal volume (V_T_) of 7 mL·kg^− 1^ predicted body weight. Ventilation frequency was set to maintain an end-tidal carbon dioxide partial pressure between 35 and 40 mmHg. In all patients, the initial PEEP was set to 5 cmH_2_O. Following these baseline measurements, the randomization was disclosed. In the control group, the PEEP was maintained for the whole procedure. In the intervention group, the PEEP was adjusted dynamically according to the recommendations resulting from the intratidal compliance profile analysis (see below).

### Gliding-SLICE

To calculate nonlinear intratidal C_RS_ profiles via the gliding-SLICE method, we chose 21 equidistant slices as a tradeoff between calculation effort and reasonable resolution. The resulting intratidal compliance curves were classified into six different compliance profiles, as described earlier [19, 20, 23]. In brief, a second-order polynomial was fitted into the compliance-volume curve, and the resulting segment of a parabola was assumed to represent the compliance-volume curve in a filtered form. If the segment showed an increase of more than 20% of the compliance maximum, the profile was classified as containing an increasing part. A segment decreasing by more than 20% of the compliance maximum was classified as containing a decreasing part. A segment containing the angular point of the parabola was classified as containing the horizontal part. A compliance profile with less than 20% change was classified as horizontal (Fig. 1) [21]. The decision support system suggested a PEEP increase of 2 cmH_2_O in the case of a merely increasing compliance profile, 1 cmH_2_O in the case of an increasing compliance profile with a horizontal component, a PEEP decrease of 2 cmH_2_O for a merely decreasing compliance profile, and 1 cmH_2_O in the case of a decreasing compliance profile with horizontal component. A merely horizontal compliance profile resulted in the suggestion to maintain PEEP as it was.

### Electrical impedance tomography

Regional ventilation was measured via electrical impedance tomography (EIT) (PulmoVista 500, Dräger Medical) every 10 min for a duration of 2 min. EIT recordings were evaluated offline using software developed in Matlab (MATLAB R2014a, The Mathworks Inc., Natick, MA, USA). As a first step, the relevant lung areas were determined for each patient by applying the lung area estimation method [24, 25] to the raw EIT data. The functional region of interest was selected by deleting all pixels with an impedance change smaller than 20% of the maximum tidal impedance change. The remaining pixels were mirrored to compensate for potential atelectatic areas. The obtained lung area was then applied to all the recorded raw EIT images. After this preprocessing, the functional impedance images were generated by subtracting the frames corresponding to the start of inspiration from the frames corresponding to the end of inspiration. These functional images (f-EIT) thus represented the distribution of the tidal volume for each breath. To assess potential changes in regional ventilation, tidal variation as well as a gain and loss calculations were performed and compared between the two groups. The gain and loss calculations were based on subtracting the functional impedance images of different time points to directly compare differences in ventilation between them. In this study, the averaged f-EIT images of the first EIT recording (baseline measurement, prior to the surgical procedure) and the averaged f-EIT images of the last EIT recording (after the surgical procedure was finished) were subtracted for each patient. The resulting differential images were split into ventral and dorsal parts and the number of positive (‘gain’) and negative (‘loss’) pixels were calculated for each such region. A gain was represented by the number of pixels that exhibited an increase in aeration in the last measured EIT sequence compared to the first (baseline) measured EIT sequence and loss was shown by a decrease in aeration. The results were compared between the two different groups. The change in tidal volume (ΔV_T_) was calculated as the difference between gain (TVG) and loss (TVL) (ΔV_T_ = TVG - TVL) for the previously defined ventral (ΔV_T,v_) and dorsal (ΔV_T,d_) lung areas. This provided a measure for changes in regional ventilation. If this difference was positive, we assumed an increase in regional ventilation in the respective lung area, whereas a negative difference indicated a decrease in regional ventilation [26].

Tidal variation (impedance distribution) is the percentage of tidal volume going to the ventral (TV_v_) and the dorsal areas (TV_d_). This was calculated for all the functional impedance images using Eq. 1,

1 $$ {TV}_v=\frac{\sum {x}_{i,v}}{\sum {x}_i}\ \mathrm{or}\ {TV}_d=\frac{\sum {x}_{i,d}}{\sum {x}_i} $$

where x_i,v_ are the impedance values in the ventral region, x_i,d_ the impedance values in the dorsal region and x_i_ the sum of all impedance values of the f-EIT under consideration. Tidal variation was calculated for each averaged f-EIT image of each 2-min EIT recording.

### End points and data collection

The frequency of each type of nonlinear intratidal C_RS_ profile (measured using the gliding-SLICE method) was the primary endpoint of this study. The secondary endpoints were regional ventilation (ventral and dorsal ventilation distribution, ventral and dorsal gain and loss and tidal variation), the respiratory system variables (peak inspiratory pressure [PIP], P_Plat_, mean tracheal pressure [P_mean_], PEEP) and hemodynamic variables (systolic blood pressure [BP_sys_], diastolic blood pressure [BP_dias_], heart rate and mean arterial pressure [MAP]). The intratidal compliance profiles, respiratory, and hemodynamic variables were recorded continuously during the study protocol. EIT measurements were performed every 10 min for a duration of 2 min.

### Sample size calculation and statistical evaluation

No data are available concerning the variance of frequencies of compliance profiles. Therefore, we based our sample size calculation on estimation of a general standardized effect size *e*, being the quotient of differences in means and standard deviation. With regard to our approach, which adapted PEEP according to the measured compliance profile, we assumed a large effect size and therefore chose e = 0.8 [27]. In regard to the trial design (unpaired test conditions) and an assumed e of 0.8, 50 patients were required to reach a test power of 0.8 with a desired level of significance of 0.05.

To compensate for potentially incomplete data sets, 60 patients were recruited. Data are presented as means (standard deviation). Differences between the two groups were assessed with the unpaired Students t-test. Statistical significance was considered for *p* < 0.05. Shapiro–Wilk tests were used to confirm that the assumed normal distribution could not be rejected. For data not normally distributed, differences between the two groups were assessed with Mann–Whitney U tests.

## Results

**Fig. 2:**
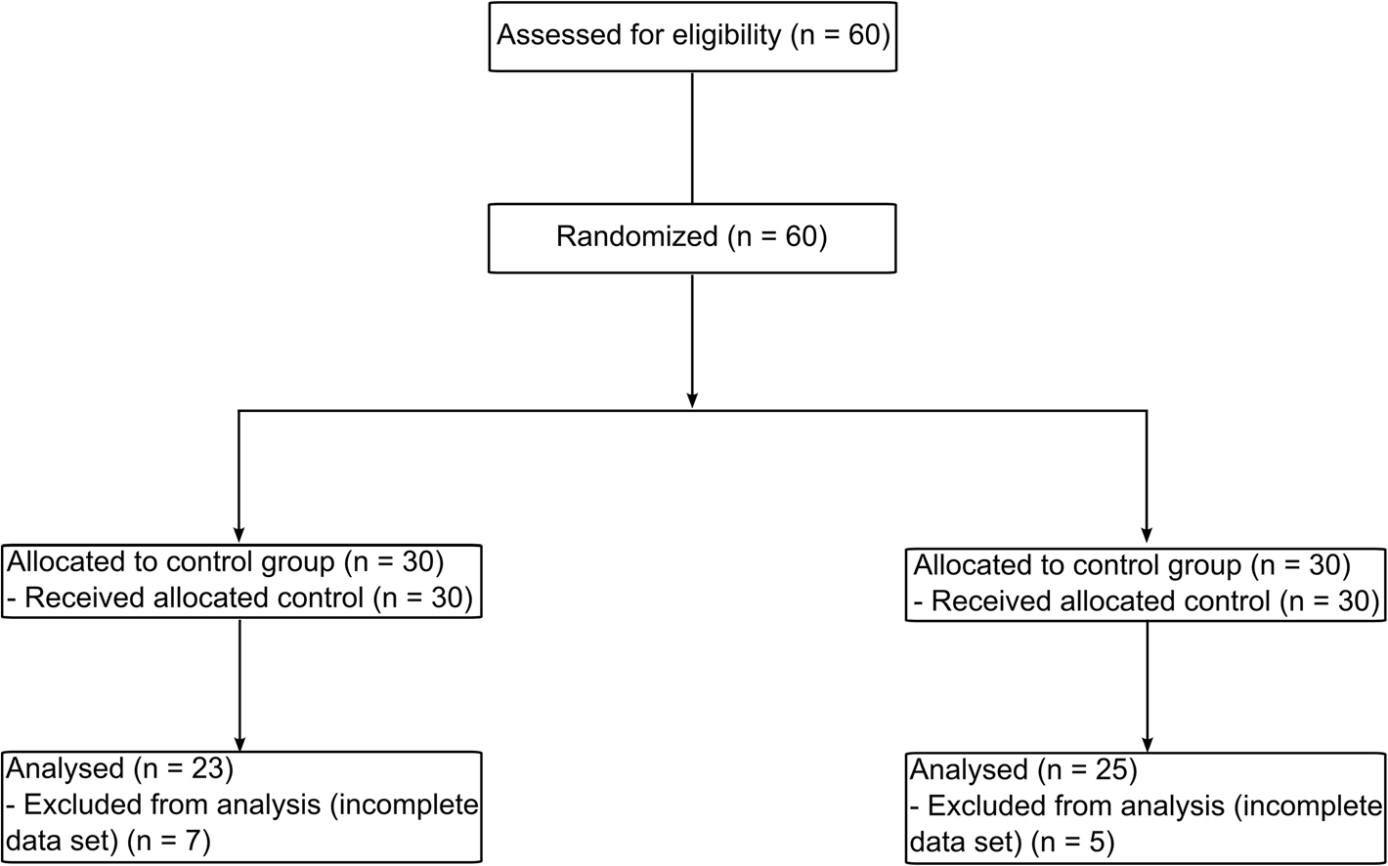
Flow diagram of the study population

Patients were enrolled from November 5, 2015 to January 29, 2016. In total, 60 patients were included. Twelve patients had to be excluded due to incomplete data sets (Fig. 2). During the study protocol, no adverse or serious events occurred. Age, gender, ASA physical status, predicted body weight, actual body weight and body mass index were comparable between the two groups (Table 1).

**Table 1:** Patients characteristics (*n* = 48)

Parameter	Control (*n* = 23)	Intervention (*n* = 25)	*p*-value
Age (yr)	50.1 (17.0)	45.0 (16.0)	0.150
Gender (n), female/male	12/11	6/19	0.226
ASA I/II/III (n)	10/12/1	8/17/0	0.506
PBW (kg)	47.4 (2.6)	48.3 (2.6)	0.491
ABW (kg)	73.7 (13.7)	79.6 (14.5)	0.249
BMI (kg·m^−2^)	24.5 (3.3)	26.5 (5.2)	0.178

*ASA* physical status according to the American Association of Anesthesiologists, *PBW* predicted body weight, *ABW* actual body weight, *BMI* body mass index. Data are expressed as mean (SD)

### Respiratory and hemodynamic variables

In 12 patients in the intervention group (48%), the PEEP was adjusted according to the intratidal compliance profile analysis. In 11 patients (44%), the PEEP was increased, as the corresponding compliance profile analysis showed increasing compliance profiles. In 7 of these patients (28%), the PEEP was thenceforward held constant. In 3 of the patients in the intervention group (12%), the PEEP was adjusted twice. In 2 patients (8%), the PEEP was adjusted three times. PEEP was higher in the intervention group compared to the control group [control: 5.0 (0.2) cmH_2_O; intervention: 5.8 (1.1) cmH_2_O, *p* < 0.001; range control: 5.0–5.0 cmH_2_O; range intervention: 3.9–8.5 cmH_2_O]. In total, PEEP was adapted in 12 patients in the intervention group (48%). These individualized PEEP adaptations had no significant effect on the other measured respiratory system or hemodynamic variables (Table 2). The frequencies of nonlinear intratidal C_RS_ profiles showed no significant difference between the two groups (Table 3).

**Table 2:** Respiratory and hemodynamic variables

Variable	Control (*n = 23*)	Intervention (*n = 25*)	*p*-value
V_T_ (mL)	541.9 (71.9)	552.6 (61.9)	0.565
V_T_ PBW (mL·kg^−1^)	7.4 (0.9)	7.1 (0.9)	0.300
VF (·min^−1^)	11.8 (1.3)	11.7 (1.7)	0.843
PIP (cmH_2_O)	16.6 (2.7)	17.1 (3.1)	0.722
P_Plat_ (cmH_2_O)	14.0 (2.3)	14.3 (2.4)	0.656
P_mean_ (cmH_2_O)	8.6 (0.9)	8.3 (0.9)	0.400
PEEP (cmH_2_O)	5.0 (0.0)	5.8 (1.1)	< 0.001
ΔP (cmH_2_O)	8.9 (2.3)	8.5 (2.0)	0.695
C_RS_ (mL·cmH_2_O^−1^)	63.2 (14.0)	67.8 (15.9)	0.508
FiO_2_	60.6 (1.6)	60.4 (1.5)	0.802
SpO_2_	99.1 (0.8)	98.8 (0.9)	0.177
PetCO_2_ (mmHg)	37.4 (1.5)	38.9 (4.6)	0.296
Heart rate (·min^−1^)	54.9 (7.8)	55.4 (9.0)	0.796
BP_sys_ (mmHg)	101.1 (10.2)	100.4 (11.6)	0.236
BP_dias_ (mmHg)	62.8 (12.5)	61.7 (12.3)	0.667
MAP (mmHg)	75.6 (11.0)	74.6 (11.1)	0.296
Duration of anesthesia (min)	83.2 (33.3)	87.5 (28.7)	0.378

*V*_*T*_ tidal volume, *V*_*T*_*PBW* tidal volume per predicted body weight, *VF* ventilation frequency, *PIP* peak inspiratory pressure, *P*_*Plat*_ plateau pressure, *P*_*mean*_ mean airway pressure, *PEEP* positive end-expiratory pressure, *ΔP* driving pressure, *C*_*RS*_ respiratory system compliance, *FiO*_*2*_ fraction of inspired oxygen, *SpO*_*2*_ peripheral oxygen saturation, *PetCO*_*2*_ end-tidal carbon dioxide partial pressure, *BP*_*sys*_ systolic blood pressure, *BP*_*dias*_ diastolic blood pressure, *MAP* mean arterial pressure. Data are expressed as mean (SD)

**Table 3:** Frequencies of compliance profiles from 48 patients

Compliance profile	Control (*n = 23*)	Intervention (*n = 25*)	*p*-value
Horizontal (%)	85.5 (28.1)	92.8 (9.6)	0.1162
Merely Increasing (%)	9.6 (20.8)	3.5 (6.4)	0.1727
Increasing-horizontal (%)	3.8 (8.5)	2.9 (4.8)	0.6626
Merely Decreasing (%)	0.2 (0.5)	0	0.4379
Horizontal-decreasing (%)	0.2 (0.8)	0.6 (1.9)	0.0797
Mixed (%)	0.7 (3.0)	0.4 (1.6)	0.6816

Differences between the two groups were assessed with Mann-Whitney U tests. Frequencies were adapted to the duration of mechanical ventilation. Data are expressed as mean (SD)

### EIT measurements

**Fig. 3:**
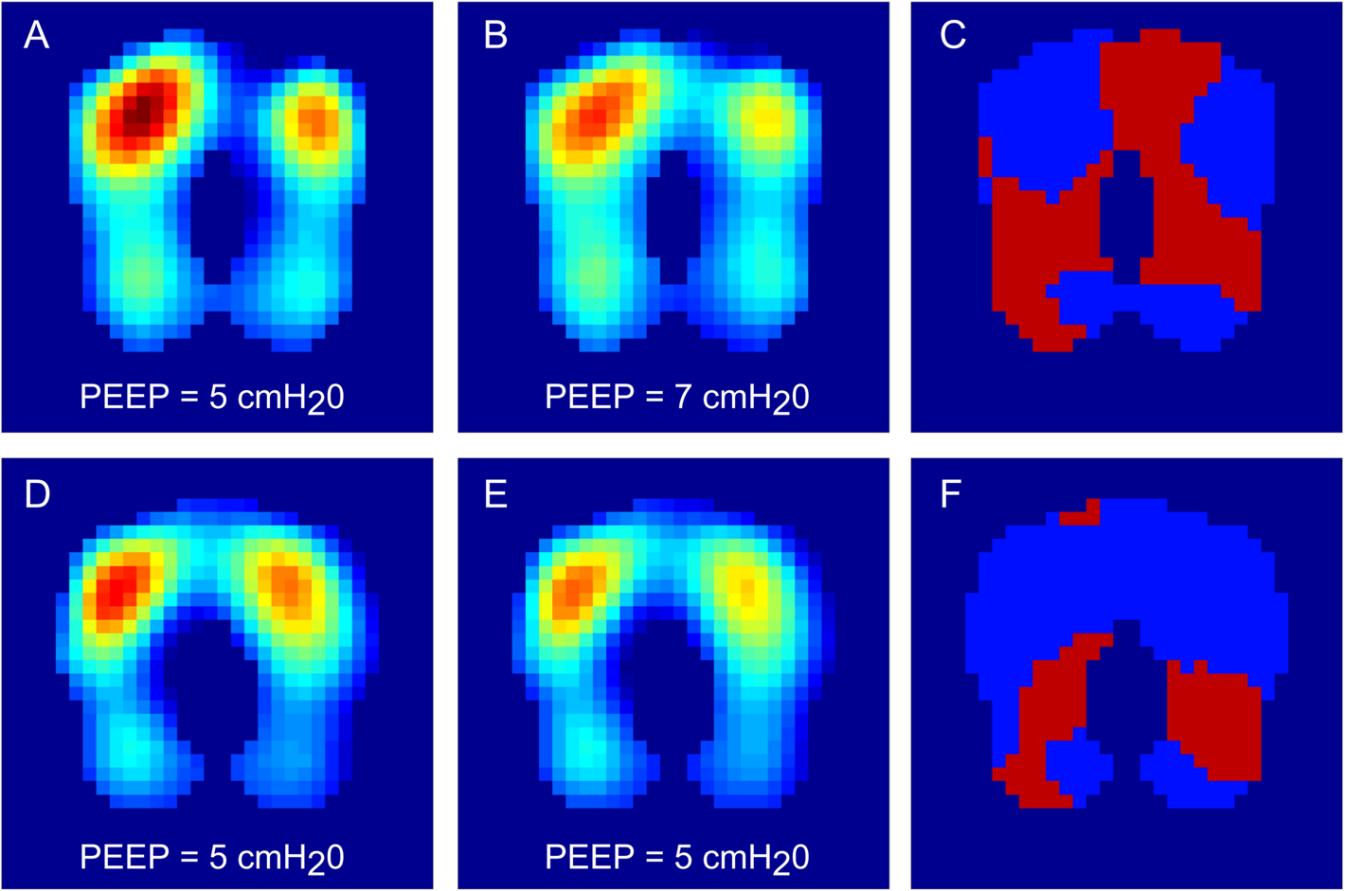
Functional impedance images (f-EIT) of two respective exemplary patients. According to the study protocol, volume-controlled ventilation was started with a PEEP of 5 cmH_2_O. In the exemplary patient in the intervention group (**a**-**c**), the PEEP was then increased to 7 cmH_2_O as the intratidal compliance profile analysis indicated a merely increasing compliance profile. In the patient in the control group (**d**-**f**), the PEEP was maintained at 5 cmH_2_O. f-EIT images were generated by subtracting the frames corresponding to the start of inspiration from the frames corresponding to the end of inspiration. **a** f-EIT image of the exemplary patient of the intervention group initially ventilated with PEEP 5 cmH_2_O; **b** f-EIT image of the exemplary patient of the intervention group during the last EIT measurement after the surgical procedure was finished; **c** Illustration of gain (red) and loss (blue) for the patient in the intervention group; **d** f-EIT image of the exemplary patient of the control group during baseline measurements; **e** f-EIT image of the exemplary patient of the control group during the last EIT measurement after the surgical procedure was finished; **f** Illustration of gain (red pixels) and loss (blue pixels) for the patient in the control group. Gain represents the amount of pixels that exhibited an increase in ventilation in the end compared to the beginning and loss the decrease in ventilation accordingly

The regional impedance distribution showed no significant difference in ventilation distribution between the two groups (Table 4). The gain and loss calculations showed a significant decrease in loss of ventral regional ventilation between the two groups [loss of ventral regional ventilation of 41.0 (16.s3)% in the control group and 29.7 (16.8)% in the intervention group, *p* = 0.039]. In the dorsal lung area, the gain in regional ventilation was higher in the intervention group [14.3 (11.9)%] than in the control group [24.6 (13.0)%, *p* = 0.013] (Fig. 3). In the intervention group, the loss of dorsal regional ventilation was less pronounced [16.4 (12.7)%] than in the control group [25.9 (13.9)%, *p* = 0.028] (Table 4). TV_v_ and TV_d_ showed no significant difference between the two groups. ΔV_T,v_ indicated a lower difference between gain and loss in the intervention than in the control group in the ventral lung area (ΔV_T,v_ [control group] = − 22.2 (31.1)%; ΔV_T,v_ [intervention group] = − 0.4 (34.2)%, *p* = 0.044). ΔV_T,d_ indicated a lower difference between gain and loss in the intervention than in the control group in the dorsal lung area (ΔV_T,d_ [control group] = − 11.6 (24.6)%; ΔV_T,d_ [intervention group] = 8.25 (25.4)%, *p* = 0.017) (Table 4).

**Table 4:** Measurements of regional ventilation

Measurements of regional ventilation	Control (*n = 23*)	Intervention (*n = 25*)	*p*-value
Gain ventral [%]	18.8 (15.5)	29.3 (17.6)	0.056
Loss ventral [%]	41.0 (16.3)	29.7 (16.8)	0.039
Gain dorsal [%]	14.3 (11.9)	24.6 (13.0)	0.013
Loss dorsal [%]	25.9 (13.8)	16.4 (12.7)	0.028
ΔV_T,v_ [%]	−22.2 (31.1)	−0.4 (34.2)	0.044
ΔV_T,d_ [%]	−11.6 (24.8)	8.25 (25.4)	0.017
TV_v_ [%]	63.9 (13.1)	60.2 (15.1)	0.368
TV_d_ [%]	36.1 (13.1)	39.8 (15.1)	0.368

Differences between the two groups were assessed with Mann-Whitney U tests. ΔV_T,v_, change in tidal volume (difference between gain and loss) for the ventral lung area; ΔV_T,d_, change in tidal volume (difference between gain and loss) for the dorsal lung area; TV_v_, percentage of tidal volume in ventral lung areas; TV_d_, percentage of tidal volume in dorsal lung areas. Data are expressed as mean (SD)

## Discussion

In this study, we compared the effects of individualized PEEP titration performed according to bedside analysis of the frequencies of nonlinear intratidal C_RS_ profiles (measured using the gliding-SLICE method). The main finding is that the individualized PEEP titration improved regional ventilation without affecting impedance distribution and the respiratory or hemodynamic variables negatively.

### Respiratory and hemodynamic variables

Besides PEEP, none of the respiratory and hemodynamic variables differed between the two patient groups. PEEP is generally associated with recruitment and one might expect that C_RS_ increases with increasing PEEP. However, in agreement with earlier studies [14, 28] C_RS_ remained unchanged. In our study, patients showed respiratory system mechanics that were mostly characterized by a horizontal compliance profile, and consequently PEEP adaptations were performed less frequently than expected. It follows that the observed improvement in regional ventilation may have increased C_RS_, if the studied patient group had included more patients with impaired respiratory system mechanics or who underwent surgical procedures associated with an increased risk for altered respiratory functions (e.g., laparoscopic surgery). Since this is the first study in which we applied individualized PEEP titration according to compliance profile analysis, we did not include patients at risk for impaired respiratory system performance. One might speculate further that the comparably high alveolar recruitment in the studied patients was the reason we did not find significant differences in C_RS_. This hypothesis can be supported by two clinical trials that provided preliminary investigations of the gliding-SLICE method [14, 28]. In both studies, lower levels of PEEP (such as 5 and 7 cmH_2_O) did not prevent from C_RS_ profiles indicating recruitment/derecruitment. In both studies, intratidal compliance profile analysis was used as a bedside measurement for predefined PEEP settings. In the present study, this analysis was used to guide PEEP titration individually. One might also speculate that the longer duration of the surgical procedure (mean duration of surgery of 120 min and 184 min vs. 83.2 min (control group) and 87.5 min (intervention group) in the present study) [14, 28] led to a more pronounced impairment of respiratory system mechanics and thus of intratidal C_RS_ profiles. In the present study, obesity was an exclusion criterion, whereas in one of the previous studies [14], obese patients were included. Further studies are needed to provide more detailed information about the impact of an individualized PEEP titration strategy based on the gliding-SLICE method on respiratory function in patients with impaired respiratory system mechanics.

By increasing the intrathoracic pressure, PEEP was shown to affect the cardiac performance by altering the left ventricular preload, afterload, and cardiac contractility [29]. Previous studies found that, in case of increasing intratidal compliance profiles, a small increase in PEEP led to ventilation with horizontal compliance [14, 28]. Since the overall increase of PEEP in our intervention group was comparably low, it is not surprising that our individualized PEEP titration had no effect on the measured hemodynamic variables.

Previously described techniques for titrating PEEP (the decremental PEEP trial [30], dead space fraction [31], indices of regional ventilation [32–34], esophageal pressure [35], or other imaging techniques [36]), require additional equipment, involve an additional burden for the patient, or may per se not be available at the bedside. The techniques based on the determination of best PEEP from static respiratory system variables, such as the static pressure-volume curve, did not contribute to the dynamic intratidal changes in respiratory system mechanics [37]. Moreover, they require a prolonged maneuver during which the patient is not sufficiently ventilated. During a decremental PEEP trial, adequate ventilation is warranted however, to identify the PEEP for maximum C_RS_, the optimal PEEP must necessarily be exceeded during the maneuver. Thus, previously described PEEP titration methods often bear the risk of overdistension and cannot be applied continuously. By contrast, PEEP titration based on the intratidal compliance profile does not require a maneuver, may be applied on a breath-by-breath analysis, and is applicable for consecutive PEEP adjustment.

### Regional ventilation

Even in patients without impaired respiratory function, induction of general anesthesia and consecutive mechanical ventilation bear the risk of atelectrauma [38]. As a noninvasive, radiation-free method, EIT can be used to monitor regional ventilation and the formation of atelectasis [39].

Comparing the baseline measurements (the EIT sequence before the surgical procedure) and the last EIT sequence (after the end of the surgical procedure) with gain and loss calculations showed a significant increase in aeration in the intervention group. This is not surprising, since PEEP was higher in the intervention group, which led to an increase in aeration [40]. The detected changes in regional gain and loss calculations might suggest that the individualized PEEP titration strategy, according to the gliding-SLICE method, reduced the loss of ventilation in the dependent lung areas. However, the detected effect was very limited; the frequencies of the compliance profiles, the TV_v_ and TV_d_ values, and the respiratory system mechanics were comparable.

Tidal variation did not differ significantly between the two groups. The larger part of ventilation remained for both groups in the ventral region of the lung at all times. Again, this is expected for mechanically ventilated patients [41]. However, one has to keep in mind that a shift in tidal variation from ventral to dorsal regions would indicate recruitment. This would be very unlikely in lung-healthy patients, since their lungs are already very well recruited.

It might seem that the results from our gain and loss calculations contradict the findings for tidal variation development. However, we found almost equal gain in both the ventral and the dorsal areas for both groups. We argue that this does not necessarily change the ventilation fraction in these parts. Consider as an example an hourglass at a certain time point when more sand is in the top compartment than in the bottom compartment. If the amount of sand in the top compartment is increased and the diameter of the connecting tube is increased accordingly, there would be more sand in both compartments, but the fraction of sand in the top compartment would not change. In contrast to this analogy, the tidal volume was held constant in both groups, but with increasing PEEP the residual capacity of the lung was increased as well [42]. We would also speculate that redistribution of volume, either based on pendelluft effects or from areas outside our observation plane, might also have contributed to the surplus in aeration.

### Limitations of the study

We did not perform arterial blood gas analyses or invasive blood pressure measurement to evaluate hemodynamic performance with a higher temporal resolution. Placing an arterial line was not part of our standard treatment for the patients included in this study. Since the intention of our study was to investigate the impact of a comparable new individualized PEEP titration strategy in a non-injured respiratory system, we did not include patients with impaired respiratory function. Based on our earlier study, we expected a large effect size, without having any data on variability of the frequencies of compliance profiles available, however. Therefore, the study was underpowered for detecting differences in frequencies of compliance profiles between the two groups. This may have been caused by our choice of an approach utilizing a general standardized effect size for the sample size calculation. This may limit the interpretation of our results.

Further studies are required to investigate the potential impact of PEEP titration based on bedside analysis of nonlinear intratidal compliance on the respiratory system mechanics in patients prone to impaired respiratory function.

## Conclusions

This is the first study to investigate regional ventilation during PEEP titration guided by intratidal compliance profile analysis in patients. Our individualized PEEP titration strategy led to an improvement in global aeration gain. Bedside analysis of the nonlinear intratidal mechanics of the respiratory system did not improve respiratory system mechanics and compliance profiles. The observed global increase in aeration indicated by the calculations of regional gain and loss and change in tidal volume might just indicate the slight increase in aeration due to the small PEEP increase in the intervention group.

## Data Availability

The datasets used and analyzed during the current study are available from the corresponding author on request. Please note that EIT data files require large memory. A separate data transfer service will be used to transfer EIT data files.

## Abbreviations

ASA: American Society of Anesthesiologists
BP_dias_: Diastolic blood pressure
BP_sys_: Systolic blood pressure
C_RS_: Compliance of the respiratory system
C_stat_: Quasi-static compliance of the respiratory system
EIT: Electrical impedance tomography
EI_Th_: Mean thoracic electrical impedance
FeO_2_: Expiratory oxygen concentration
FiO_2_: Inspiratory oxygen concentration
I:E: Ratio of inspiratory time to expiratory time
MAP: Mean arterial pressure
PEEP: Positive end-expiratory pressure
PIP: Peak inspiratory pressure
P_mean_: Mean airway pressure
P_Plat_: Plateau pressure
SpO_2_: Peripheral oxygen saturation (pulse oximetry)
VF: Ventilation frequency
V_T_: Tidal volume
